# Death from Inhalation of Chloroform

**Published:** 1873-10

**Authors:** W. H. Mussey

**Affiliations:** Professor of Surgery, Miami Medical College


### Selections.

### DEATH FROM INHALATION OF CHLOROFORM.

BY W. H. MUSSEY, M. D., *Professor of Surgery, Miami Medical College.*

Adolph Hummler, cooper, aged 29, German by birth, called on me August 6th, with the terminal phalanx of right thumb hanging by a slight thread of skin on the dorsal aspect, having received an injury that exposed the next phalanx. As there was too much mangling of the detached portion to admit of osteoplasty, I proposed to dissect back the integument, and remove a sufficient portion of the protruding bone to make a good stump.

The patient insisted upon inhaling chloroform, notwithstanding my unwillingness to give it, as the operation was a slight one, and as I had no assistant then in the office ; but I yielded to his entreaties, gave to him two ounces of brandy in water, and commenced the administration of the anæsthetic at about five minutes before noon. The patient took it satisfactorily for two minutes, when my nephew, Dr. R. D. Mussey, came in, and I resigned the further administration into his hands. Soon, the patient stated to his brother, in the German language, that "they were making him drunk," and immediately commenced to struggle and slide down on the couch on which he was lying. The chloroform was removed, and the patient replaced in his former position, at which moment he had a convulsion ; the skin of his chest, neck and face became purple, and the breathing profoundly stertorous. As the clock struck the noon hour, the breathing ceased.

I immediately instituted artificial respiration, by inflating the lungs from my own, and by pressure upon the chest,—every other filling of the lungs being from the air of the room. I placed forceps upon the tongue and drew it out, though there was no obstruction to the ingress of air, nor was there the dropping back of the tongue. For six or seven minutes I persisted in this method of artificial respiration, when I determined to make use of galvanism, and directed Dr. R. D. Mussey to make ready the constant-current battery, and myself prepared the Faradaic battery. The first-named current was applied to the phrenic nerves, the current being broken every two or three seconds, regularly and irregularly ; then the negative electrode was applied to the phrenic nerves, and and the positive to the diaphragm. Then the Faradaic battery was applied to the positions above named, and artificial respiration maintained by strong pressure of the electrode over the region of the heart, and its sudden removal. This was satisfactory, so far as its action was concerned, and was continued five minutes. Not perceiving any beneficial results from this process, inflation of the lungs was repeated, and this form of artificial respiration was tried for a few minutes. All efforts proved unavailing, and, after persisting about twenty minutes in our attempts to resuscitate, we were compelled to admit that the man was dead.

By order of the coroner, at 8½ P. M. an autopsy was made by H. A. Clark, M. D., an entire stranger to me, who politely invited me to be present. N. P. Dandridge, M. D., Pathologist, Cincinnati Hospital, "assisted" in the autopsy, by making the entire dissection with his usual celerity.

After the taking of testimony, August 7th, this record of the autopsy was presented by Dr. Clark.

"Cincinnati, August 7th, 1873.

"In compliance with the request of Dr. P. F. Maley, coroner of this county, I made a post-mortem examination of the body of Adolph B. Hummler, at the corner of Abigail and Main streets, at 8½ o'clock P. M., on the 6th instant. The body presented a healthy appearance ; was well formed, and in good condition. The thumb of the right hand was nearly severed by a lacerated wound just above the last joint, being merely held by a piece of skin. There was a contused wound of the temple, just above the outer corner of the right eye, and involved only the skin. There was the mark of another contused wound on the right side of the head, above the ear. These wounds of the head had produced no obvious effect upon the brain. Upon opening the body, the lungs were found slightly congested, but otherwise healthy. Mucous membrane of windpipe slightly reddened. The heart was found empty; left ventricle contracted, right ventricle flaccid. All the valves were perfectly normal. The blood was in a fluid condition; stomach, liver and intestines healthy. The kidneys were uniformly congested, but not to a very marked degree; other urinary organs healthy. The brain, with its membrane and arteries, presented no abnormal appearance. The quantity of cerebro-spinal fluid was larger than usual.

H. A. Clark, M. D."

In the testimony before the coroner's jury, the wife says: "We were married eight years. My husband was a temperate man; drank five or six glasses of beer daily since our marriage." The next witness, Joseph Siefert, testified: "I have known deceased three years; he drank whisky every morning regularly; he was a temperate man."

I submit, if the admission of the wife to the daily use of five or six glasses of beer, and, by a boon companion, of a drink of whisky every morning, does not justify the inference that the deceased was a genuine soaker, and used much larger quantities than his wife knew of.

The last-named witness told me, privately, that Hummler fell through a hatchway two or three weeks ago, and struck upon his head, "when he had too much on;" that he had not been really well since; and that, in handling some tools, he accidentally struck his face, over the external orbit of left eye. How much injury to the brain these external blows may have caused, is not clear. They may have had important relations with the causes of death.

In the autopsy, there was noticed, on the opening of the dura mater, an unusual quantity of serum—one ounce, I should think; also, under the arachnoid there was a preternatural quantity, though there was no unusual redness present. The skull was one-third of an inch in thickness, of uniform density, (no diploe,) very much like a syphilitic hypertrophy. There was decided congestion of the kidneys.

The administration of the anæsthetic was by putting a sponge into the mouth of a one-ounce phial, and shaking a dozen drops at a time upon one thickness of a linen handkerchief held loosely over the face ; and only one-third of an ounce of chloroform was used.

The chloroform caused the death. The ultimate conditions preparing the patient for the fatal issue are matters of speculation. Without doubt, alcoholism had its part of the responsibility. Natural or acquired idiosycracies may be supposed. The patient died with *coma*, an unfrequent form of death from anæsthetics ; in most cases there being asphyxia or syncope

I have administered anæsthetics for a period of twenty-eight years, as often as any one in full practice,—with one fatal result. How many have administered opium or its compounds,—veratrum, alcohol and other poisonous drugs,—to the same extent, and have had but ONE death from the article administered ?

